# The impact of single nucleotide polymorphism in monomeric alpha-amylase inhibitor genes from wild emmer wheat, primarily from Israel and Golan

**DOI:** 10.1186/1471-2148-10-170

**Published:** 2010-06-09

**Authors:** Ji-Rui Wang, Yu-Ming Wei, Mei Deng, Eviatar Nevo, Ze-Hong Yan, You-Liang Zheng

**Affiliations:** 1 Triticeae Research Institute, Sichuan Agricultural University, Yaan, Sichuan 625014, China; 2 Institute of Evolution, University of Haifa, Mt. Carmel, Haifa 31905, Israel; 3 Key Laboratory of Crop Genetic Resources and Improvement in Southwest China, Ministry of Education, Sichuan Agricultural University, Yaan, Sichuan 625014, China

## Abstract

**Background:**

Various enzyme inhibitors act on key insect gut digestive hydrolases, including alpha-amylases and proteinases. Alpha-amylase inhibitors have been widely investigated for their possible use in strengthening a plant's defense against insects that are highly dependent on starch as an energy source. We attempted to unravel the diversity of monomeric alpha-amylase inhibitor genes of Israeli and Golan Heights' wild emmer wheat with different ecological factors (e.g., geography, water, and temperature). Population methods that analyze the nature and frequency of allele diversity within a species and the codon analysis method (comparing patterns of synonymous and non-synonymous changes in protein coding sequences) were used to detect natural selection.

**Results:**

Three hundred and forty-eight sequences encoding monomeric alpha-amylase inhibitors (WMAI) were obtained from 14 populations of wild emmer wheat. The frequency of SNPs in WMAI genes was 1 out of 16.3 bases, where 28 SNPs were detected in the coding sequence. The results of purifying and the positive selection hypothesis (p < 0.05) showed that the sequences of WMAI were contributed by both natural selection and co-evolution, which ensured conservation of protein function and inhibition against diverse insect amylases. The majority of amino acid substitutions occurred at the C-terminal (positive selection domain), which ensured the stability of WMAI. SNPs in this gene could be classified into several categories associated with water, temperature, and geographic factors, respectively.

**Conclusions:**

Great diversity at the WMAI locus, both between and within populations, was detected in the populations of wild emmer wheat. It was revealed that WMAI were naturally selected for across populations by a ratio of dN/dS as expected. Ecological factors, singly or in combination, explained a significant proportion of the variations in the SNPs. A sharp genetic divergence over very short geographic distances compared to a small genetic divergence between large geographic distances also suggested that the SNPs were subjected to natural selection, and ecological factors had an important evolutionary role in polymorphisms at this locus. According to population and codon analysis, these results suggested that monomeric alpha-amylase inhibitors are adaptively selected under different environmental conditions.

## Background

Two major classes of methods are currently in use to detect natural selection: population methods, based on analyzing the nature and frequency of allele diversity within a species, and codon analysis methods, based on comparing patterns of synonymous and non-synonymous changes in protein coding sequences. A substantial private and public effort has been undertaken to characterize SNPs tightly associated with genetic diversity. SNPs are identified in ESTs, thus polymorphism could be directly used to map functional and expressed genes [1]. The majority of SNPs in coding regions (cSNPs) are single-base substitutions, which may or may not result in amino acid changes. However, some SNPs may alter a functionally important amino acid residue, and these are of interest for their potential links with phenotypes [2]. If the phenotypic effect impacts survival and reproduction, natural selection operates on SNP alleles [3]. Evolutionary pressures of various kinds have often been hypothesized to cause active and rapid evolutionary changes. Positive selection is a form of natural selection that influences the process by which new advantageous genetic variants sweep across populations. Though advantageous mutations are of great interest, they are difficult to detect and analyze because neutral and deleterious mutations predominate by frequency. In contrast, purifying selection is expected to act against mutations that have deleterious effects on protein structure by causing changes to functionally important amino acid residues or by altering the regulation of gene expression [4]. Since SNPs are almost always bi-allelic, relatively low-gene diversity at a given SNP site is equivalent to lower allelic frequency than the less frequent of the two alleles. The reduction of gene diversity at these SNP sites, in comparison to SNPs in the same genes that do not affect protein structure, provides evidence that the purifying selection has reduced the population allelic frequencies of deleterious SNP alleles [5]. A classic measure for selective pressure on protein-coding genes is the dN/dS (Ka/Ks) ratio. The ratio of the observed non-synonymous mutation rate to the synonymous mutation rate can be utilized as an estimate of selective pressure, where dN/dS < 1 suggests that most amino acid substitutions have been eliminated by the purifying selection, while a dN/dS > 1 indicates positive selection [6].

Wild emmer wheat (*Triticum dicoccoides*) presumably adaptively diversified from northeastern Israel and Syria into the Near East Fertile Crescent, where it harbors rich genetic diversity and resources [7]. Previous studies in cereals have shown significant nonrandom adaptive molecular genetic differentiation at single and multi-locus structures among micro-ecological environments [8,9]. The genetic differentiation of variable wild emmer wheat populations included regional and local patterns with sharp genetic differentiation over short distances [10].

Alpha-amylase inhibitors are attractive candidates for the control of seed weevils as these insects are highly dependent on starch as an energy source [11]. *In vitro *and *in vivo *trials using α-amylase inhibitors, including those made in field conditions, have now fully confirmed their potential for increasing yields by controlling insect populations [11]. In cereal seeds, α-amylase inhibitor proteins with 120-130 amino acids, which include trypsin inhibitors as well as α-amylase inhibitors, can be grouped into one large family on the basis of the homology between their amino acid sequences [12]. It is known that the bulk of wheat albumins consists of a few amylase isoinhibitor families that are very likely phylogenetically related and coded by a small number of parental genes [13]. The monomeric, homo-dimeric, and hetero-tetrameric α-amylase inhibitors (WMAI, WDAI, and WTAI) are the main members of a family of wheat kernel proteinaceous inhibitors that are active on exogenous alpha-amylases of various origins. WMAI-0.28, WMAI-0.39, WDAI-0.19, and WDAI-0.53 were extensively investigated [14,15]. WMAI is a proteinaceous inhibitor with a molecular weight of 12 kDa; WDAI is a 24 kDa protein formed by the combination of two 12 kDa subunits; WTAI is a mixture (about 60 kDa) of WTAI-CM2 plus 2 WTAI-CM3 plus WTAI-CM16, where none of the subunits is active on its own [14,16]. It was well established that each family was closely related, with largely identical amino acid sequences and conformational structures, and it was suggested that the inhibitors derived from common ancestral genes [17]. In a co-evolving system of plant-insect interactions, plants synthesize a variety of toxic proteinaceous and non-proteinaceous molecules for their protection against insects [18,19]. Proteinase inhibitors are therefore a potential model system in which to study basic evolutionary processes, such as functional diversification [20]. The structure and diversity of WDAI genes in wild emmer wheat from Israel was revealed, and the relationship between the emmer wheat genes and ecological factors was elucidated by 16 specific SNP markers [21]. It was found that the populations of wild emmer wheat showed a wide range of diversity in WDAI, both between and within populations.

In this study, SNP diversity of the wheat monomeric α-amylase inhibitor formed 14 natural wild emmer wheat populations in Israel and Golan. A population analysis including an examination of its ecological characteristics as well as comparing patterns of synonymous and non-synonymous changes in coding sequences of all accessions were also used to detect the natural selection of genes. The results yielded further insight into the correlation between plant defense proteinaceous inhibitors and their environmental stresses.

## Results

### Characterization of monomeric α-amylase inhibitors

Genomic PCR amplifications were conducted by specific WMAI cloning primers, and desired PCR products were detected in accessions of wild emmer wheat. A total of 348 novel gene sequences of WMAI were obtained and submitted to NCBI [GenBank: FJ874277-FJ874629], consisting of 456 bp including a 90 bp signal peptide coding domain. The frequency of SNPs was 1 out of 16.3 bases in which 28 cSNPs were detected in the coding sequence. Some population-specific SNPs were detected, such as 96G, which was found only in six sequences from the population of Daliyya (29). All of the 31 sequences from the population of Amirim (24) were 114G, 228C, 246G, 282C, 288G, 297A, 315A, 339A, and 438G while sequences from other populations had SNPs in these sites (Table 1).

**Table 1:** SNP mining and haplotype classification of WMAI from wild emmer wheat.

Haplotype																													Numbers														
SNP Position	051	072	084	096	120	**139**	141	180	183	228	**232**	246	282	288	297	315	336	**338**	**339**	**350**	**382**	396	**415**	**433**	438	**445**	**451**	**452**	Population	5	8	9	11	16	17	18	19	23	24	25	28	29	30
			
																																											
H01	G	T	C	T	G	G	A	G	C	G	G	A	T	T	G	G	C	G	G	T	T	T	A	T	C	C	G	T	4			1						1		1		1	
H02	G	T	C	T	G	G	A	G	C	G	G	A	T	T	G	G	C	G	G	T	T	T	A	T	C	C	G	C	18	1	1		2	1	5		1	2		2	2		1
H03	G	T	C	T	G	G	A	G	C	G	G	A	T	T	G	G	C	G	G	T	T	T	A	T	C	A	G	C	1											1			
H04	G	T	C	T	G	G	A	G	C	G	G	A	T	T	G	G	C	G	G	T	T	T	A	T	C	C	A	C	13		1	1	2		1			1		3	4		
H05	G	T	C	T	G	G	A	G	C	G	G	A	T	T	G	G	C	G	G	T	T	T	A	T	G	C	A	C	1												1		
H06	G	T	C	T	G	G	A	G	C	G	G	A	T	T	G	G	C	G	G	T	T	T	A	C	C	C	G	C	1						1								
H07	G	T	A	T	G	G	A	G	C	G	G	A	T	T	G	G	C	G	G	T	T	C	A	T	C	C	A	C	1			1											
H08	G	T	C	T	G	G	A	G	C	G	G	A	T	T	G	G	C	G	G	T	T	T	A	C	C	A	G	C	1							1							
H09	G	T	C	T	G	G	A	G	C	G	G	A	T	T	G	G	C	G	G	T	C	T	A	C	C	C	A	C	1														1
H10	G	C	T	T	C	G	G	G	C	C	G	G	C	G	A	A	T	G	A	T	T	T	G	T	G	C	A	C	46	14	8	7	6		2	3			1	1	1		3
H11	G	C	T	T	C	G	G	G	C	C	G	G	C	G	A	A	T	G	A	C	T	T	G	T	G	C	A	C	1								1						
H12	G	C	T	T	C	G	G	G	C	C	G	G	C	G	A	A	T	G	A	T	T	T	G	T	G	A	A	C	1								1						
H13	G	C	T	T	C	G	G	T	C	C	G	G	C	G	A	A	T	G	A	T	T	T	G	T	G	C	A	C	1			1											
H14	G	C	T	T	C	G	G	T	C	C	G	G	C	G	A	A	T	G	A	C	T	T	G	T	G	C	A	C	2			2											
H15	G	C	T	T	C	G	G	G	C	C	G	G	C	G	A	A	T	G	A	T	T	T	G	T	G	C	G	C	15	4	4	4		1					2				
H16	G	C	T	T	C	G	G	G	C	C	G	G	C	G	A	A	T	G	A	T	T	T	G	T	G	C	G	T	158	12	5	7	2	7		23	22	21	21	5	8	7	18
H17	G	C	T	T	C	G	G	G	C	C	G	G	C	G	G	A	T	G	A	T	T	T	G	T	G	C	G	T	1								1						
H18	G	C	T	T	C	G	G	G	C	C	G	G	C	G	A	A	T	G	A	T	T	.	G	T	G	C	G	T	4		1	1					2						
H19	G	C	T	T	C	G	G	G	C	C	G	G	C	G	A	A	T	G	A	T	T	T	G	C	G	C	G	T	1	1													
H20	G	C	T	T	C	G	G	G	C	C	G	G	C	G	A	A	T	G	A	T	T	T	G	T	G	A	G	C	1			1											
H21	G	C	T	T	C	G	G	T	C	C	G	G	C	G	A	A	T	G	A	T	C	T	G	T	G	C	A	C	4					2							1		1
H22	G	C	T	T	C	G	G	T	C	C	G	G	C	G	A	A	T	G	A	T	C	T	G	T	G	A	A	C	1												1		
H23	G	C	T	T	C	G	G	T	C	C	G	G	C	G	A	A	T	G	A	T	C	T	G	T	G	C	.	C	1														1
H24	G	C	C	T	C	G	G	G	C	C	G	G	C	G	A	A	T	G	A	T	T	T	G	T	G	C	A	C	1				1										
H25	G	C	T	T	C	G	G	G	C	C	G	G	C	G	A	A	C	G	A	T	T	T	A	T	C	C	A	C	1					1									
H26	G	C	C	T	C	A	G	G	A	C	A	G	C	G	A	A	C	T	A	T	T	T	A	T	G	C	A	C	10	2	3	2	1			2							
H27	G	C	C	T	C	A	G	G	A	C	A	G	C	G	A	A	C	T	A	T	T	T	A	T	G	C	G	C	2		1				1								
H28	G	C	C	T	C	A	G	G	A	C	A	G	C	G	A	A	C	T	A	T	T	T	A	T	G	C	A	T	1							1							
H29	G	C	C	T	C	A	G	G	A	C	A	G	C	G	A	G	C	T	A	T	T	T	A	T	G	C	A	C	1		1												
H30	A	C	C	T	C	A	G	G	A	C	A	G	C	G	A	A	C	T	A	T	T	T	A	T	G	C	G	T	46					10	1			1	7	14	12	1	
H31	A	C	C	T	C	A	G	G	A	C	A	G	C	G	A	A	C	T	A	T	T	C	A	T	G	C	G	T	1											1			
H32	A	C	C	T	C	A	G	G	A	C	A	G	C	G	A	A	C	T	A	C	T	T	A	T	G	C	G	T	1												1		
H33	A	C	C	G	C	A	G	G	C	C	G	G	C	G	A	A	C	T	A	T	T	T	A	T	G	C	G	T	6													6	

SNPs responsible for amino acid substitution are in bold.

**Figure 1:**
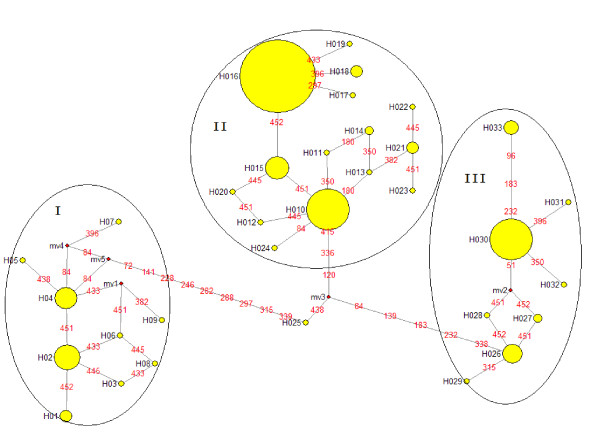
**Median-joining networks of the haplotypes of monomeric α-amylase inhibitor genes**.

**Figure 2:**
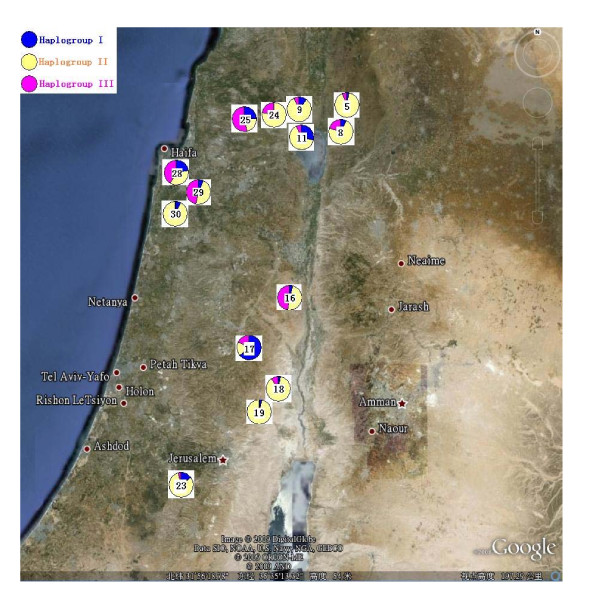
**Geographic distribution of the populations of wild emmer wheat and the three haplogroups' distribution of WMAI**. Details on numbered populations can be found in Table 7.

Thirty-three haplotypes were revealed by the sequence alignment of monomeric α-amylase inhibitor sequences from wild emmer wheat (Table 1). Haplotypes were highly separated by Median-joining network analysis, and at least three groups emerged (Figure 1). For each haplogroup, there was a primary haplotype (H02, H16, or H30), while haplotype H16 was the main one occurring in 158 WMAI sequences, followed by haplotype H10, H30, and H02. The mutations (different SNPs) between haplotypes and the primary haplotype in each group were less than five (Figure 1). Our findings indicate that the three haplogroups were not equally distributed (Figure 2). Besides the haplotypes having only one sequence, some haplotypes were characterized geographically. Fifteen sequences belonging to H15 were from the Qazrin (5), Gamla (8), Rosh-Pinna (9), Mt. Gilboa (16), and Amirim (24) populations, which were located in northern sites of Israel and Golan.

It was found that the SNPs in α-amylase inhibitor gene sequences could determine amino acid substitution in proteins (Table 2). Only nine SNPs in the nucleotide sequence of WMAI resulted in amino acid variations; among these SNPs, two were at the third codon position, and, as expected, most SNPs that resulted in amino acid changes were found at the first or second position (Table 2). Amino acid sequence alignments indicated that alpha-amylase inhibitors were highly homologous and belonged to a monomeric α-amylase inhibitor family including WMAI-0.28 and WMAI-0.39 (data not shown); however, some haplotypes shared the same deduced protein sequence. The amino acid substitutions probably determined a charge difference resulting in different relative mobility on gel electrophoresis and in differential inhibitory activities of WMAI-0.28, WMAI-0.32, WMAI-0.35, WMAI-0.39, and WMAI-0.48 with similar molecular weights [13]. Additionally, ten Cys were observed in most of the deduced proteins of the monomeric α-amylase inhibitors, and all cereal-type α-amylase inhibitors had ten Cys as well. Only seven sequences belonging to haplotypes H09, H21, H22, and H23 had Arg128 in place of Cys128 (Table 2). Interestingly, these seven sequences were all from middle Israeli populations: Mt. Gilboa (16), Beit-Oren (28), and Bat-Shelomo (30), respectively.

**Table 2:** Variation of amino acids caused by nucleotide changes in genes

Site	Substitution	Amino acid position*	Amino acid variation
WMAI-139	**A**CA-**G**CA	47	Thr-Ala
WMAI-232	**G**AC-**A**AC	78	Asp-Asn
WMAI-338-339	C**GA**-C**GG**-C**TA**	113	Arg-Arg-Leu
WMAI-350	A**T**G-A**C**G	117	Met-Thr
WMAI-382	**T**GC-**C**GC	128	Cys-Arg
WMAI-415	**G**GA-**A**GA	139	Gly-Arg
WMAI-433	**T**GG-**C**GG	145	Trp-Arg
WMAI-445	**C**CG-**A**CG	149	Pro-Thr
WMAI-451-452	**GT**C-**GC**C-**AT**C-**AC**C	151	Val-Ala-Ile-Thr

* Included the signal peptide

### Adaptive evolution analysis

The dN/dS ratio is widely used as an indicator of natural selection in gene sequences. An excess of non-synonymous mutations relative to synonymous mutations is a clear indication of positive selection, whereas a lack of non-synonymous mutations relative to synonymous polymorphisms suggests negative or purifying selection imposed by functional constraint. To analyze the natural selection of wild emmer wheat monomeric α-amylase inhibitors, the dN/dS codon analysis for purifying and positive selection was calculated.

At first, the software PAL2NAL was used to calculate the dN/dS ratio for each haplotype compared with the main haplotype, which was used as a reference (Table 3). The site-specific models implemented in codeml indicated that both purifying and positive selection had occurred; however, the purifying selection predominated. Since the main haplotype was used instead of the original sequence, other methods should be applied to clarify the selection of this protein.

**Table 3:** The calculation of synonymous and non-synonymous substitution rates for α-amylase inhibitor genes between main haplotype and other haplotypes by PAL2NAL in PAML codeml program.

Haplotype	dS	dN	dN/dS
H01	0.1560	0.0114	0.0733
H02	0.1656	0.0064	0.0384
H03	0.1670	0.0095	0.0571
H04	0.1657	0.0096	0.0577
H05	0.1505	0.0096	0.0639
H06	0.1644	0.0096	0.0583
H07	0.1682	0.0127	0.0758
H08	0.1656	0.0128	0.0771
H09	0.1640	0.0161	0.0982
H10	0.0001	0.0066	**> > 1**
H11	0.0001	0.0099	**> > 1**
H12	0.0001	0.0096	**> > 1**
H13	0.0114	0.0064	0.5583
H14	0.0111	0.0097	0.8719
H15	0.0000	0.0033	NA
H17	0.0102	0.0058	0.5741
H18	0.0101	0.0058	0.5797
H19	0.0001	0.0088	**> > 1**
H20	0.0001	0.0063	**> > 1**
H21	0.0223	0.0130	0.5824
H22	0.0116	0.0127	**1.1023**
H23	0.0113	0.0064	0.5631
H24	0.0106	0.0065	0.6207
H25	0.0356	0.0095	0.2665
H26	0.0480	0.0192	0.3998
H27	0.0482	0.0159	0.3295
H28	0.0485	0.0159	0.3274
H29	0.0611	0.0191	0.3135
H30	0.0560	0.0173	0.3086
H31	0.0679	0.0173	0.2545
H32	0.0555	0.0203	0.3652
H33	0.0575	0.0142	0.2480

Next, the selection Z-test was carried out using MEGA 3.1. To determine whether natural selection contributes to diversity in WMAI, the ratio of non-synonymous to synonymous substitutions was evaluated for each pair of haplotypes. The Tajima's Neutrality test showed that pS (segregating sites per site) = 0.06, pi (Nucleotide/amino acid diversity) = 0.02, and D = 0.82. The Z-test results revealed a very high proportion of sequences that were under selection across populations; nine haplotype sequences showed the ratio of dN/dS expected under the hypothesis of selection (dN≠dS, p < 0.05). These nine sequences represent cases where a haplotype differed from the other 32 haplotypes by more than 70%. The results of purifying and positive selection hypothesis also showed that the sequences of WMAI were contributed by both selection hypotheses in this protein (data not shown).

**Figure 3:**
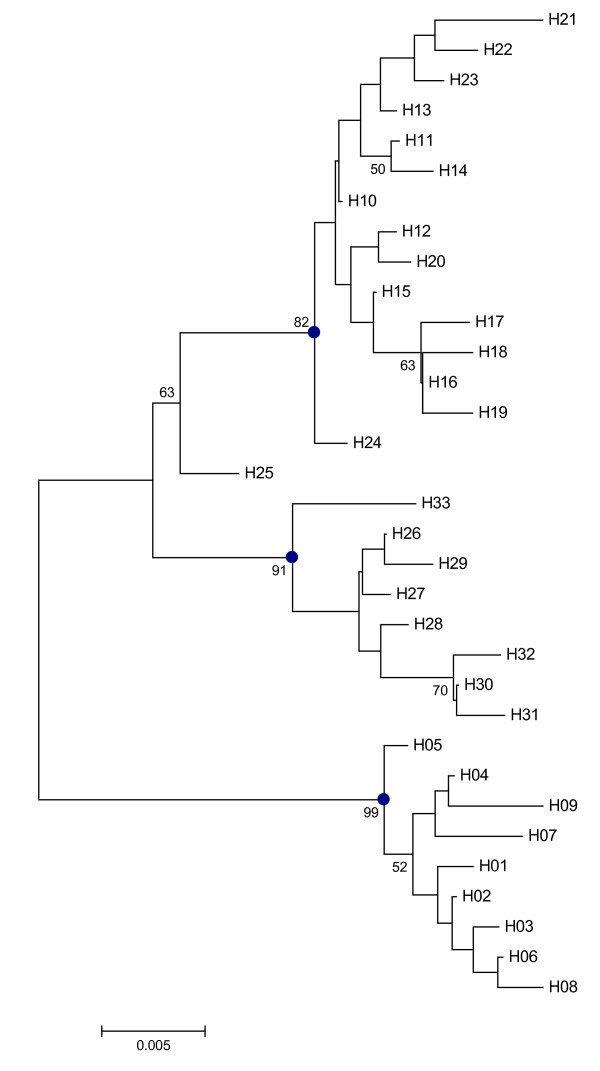
**The NJ phylogeny tree of the haplotypes used to calculate the dN/dS (ω) ratio for each site**.

Finally, we assessed positive selection with the FEL routines by HYPHY [22]. The sequence alignments and NJ tree were used to calculate the dN/dS (ω) ratio for each site (Figure 3). The results from HYPHY analyses confirm the occurrence of selection in WMAI sequences (Table 4). According to the site-by-site LRT data, it was indicated that the majority of selected amino acid residues were subjected to purifying selection. The ratio values of whole sequences were also substantially lower than 1, indicating that the inhibitors were under strong purifying selection pressure. However, few amino acid residues at the C-terminal were positively selected, suggesting that this part of the protein was subjected to directional or diversifying selection.

**Table 4:** Selection analysis of emmer wheat monomeric α-amylase inhibitor genes by HYPHY.

MODEL (Number and Description)	dN/dS	Log likelihood	Parameters
M0. Single Rate Model	0.3327	-1031.966056	C = 0.332
M1. Neutral	0.1552	-1006.740774	P = 0.844; ω = 0
M2. Selection	0.3213	-987.329667	P1 = 0.821; P2 = 0.961; ω1 = 0; ω2 = 21.860
M3. Discrete	0.3825	-985.821456	P1 = 0.909; P2 = 0.926; R_1 = 0.019; R_2 = 11.431; ω1 = 2.148
M4. Freqs	0.2055	-996.414797	P1 = 0.794; P2 = 0.393; P3 = 0.673; P4 = 0
M7. Beta	0.3808	-1018.108762	βQ = 85; βP = 44
M8. Beta and w	0.1550	-1006.746016	P = 0.845; ω = 1
M9. Beta and Gamma	0.3889	-1019.118303	α = 0.372; β = 1.119; βQ = 85; βP = 0.050
M10. Beta and (Gamma+1)	0.3932	-1018.209199	α = 0.018; P = 1; β = 0.018
M11. Beta and (Normal > 1)	0.3921	-1018.744583	P = 1
M12. 0 and 2 (Normal > 1)	0.5639	-994.433161	P = 0.894; P1 = 0.544
M13. 3 Normal	0.3967	-1018.750238	P = 0.981; P1 = 0.282

### Genetic diversity and distance of α-amylase inhibitor genes

The proportion of polymorphic loci *P *(5%), the expected heterozygosity *He*, and Shannon's information index *I *of the 14 populations of wild emmer wheat were summarized in Table 5. It was obvious that some populations had higher diversities. The values of *He *ranged from 0.094 to 0.353 with the population of Beit-Oren (28) having the highest value (0.353), followed by the population of Nahef (25). The population from Kokhav Hashahar (19) had the lowest *He *value of 0.094. Genetic distances (*D*) were calculated for comparisons of all populations based on the gene sequences of monomeric amylase inhibitors among all population pairs (Table 6). However, low *D *values ( < 0.050) were observed between some populations from different areas, and the estimates of *D *values were significantly and geographically independent. Large genetic distances and sharp genetic differentiation over long geographic distances could be found.

**Table 5:** Genetic diversity of wheat α-amylase inhibitor genes based on SNPs in populations of wild emmer wheat.

Population (No.)	*P*	*He *(St. Dev)	*I *(St. Dev)
Qazrin (5)	0.750	0.111 (0.118)	0.201 (0.170)
Gamla (8)	0.786	0.197 (0.171)	0.314 (0.239)
Rosh-Pinna (9)	0.857	0.245 (0.147)	0.390 (0.216)
Tabigha (11)	0.679	0.291 (0.231)	0.415 (0.317)
Mt. Gilboa (16)	0.821	0.248 (0.179)	0.382 (0.244)
Mt. Gerizim (17)	0.786	0.332 (0.193)	0.479 (0.270)
Gitit (18)	0.786	0.164 (0.107)	0.282 (0.171)
Kokhav Hashahar (19)	0.679	0.094 (0.078)	0.178 (0.137)
J'aba (23)	0.750	0.169 (0.119)	0.285 (0.188)
Amirim (24)	0.393	0.110 (0.144)	0.177 (0.229)
Nahef (25)	0.821	0.335 (0.187)	0.486 (0.259)
Beit-Oren (28)	0.929	0.353 (0.152)	0.519 (0.203)
Daliyya (29)	0.750	0.254 (0.180)	0.386 (0.253)
Bat-Shelomo (30)	0.679	0.120 (0.131)	0.209 (0.192)
Mean		0.261 (0.138)	0.415 (0.185)

*P*, Polymorphic per population; *He*, Expected heterozygosity (Nei's gene diversity); *I*, Shannon's information index

**Table 6:** Nei's genetic distance of inhibitor genes in 14 populations.

Population	5	8	9	11	16	17	18	19	23	24	25	28	29
8	0.0154												
9	0.0148	0.0227											
11	0.1252	0.1042	0.0644										
16	0.0370	0.0234	0.0454	0.1318									
17	0.1610	0.1044	0.1021	0.0377	0.1022								
18	0.0116	0.0313	0.0184	0.1220	0.0300	0.1463							
19	0.0142	0.0441	0.0273	0.1546	0.0426	**0.1917**	0.0037						
23	0.0258	0.0445	0.0286	0.1227	0.0286	0.1393	0.0044	0.0075					
24	0.0242	0.0366	0.0465	**0.1819**	0.0190	0.1739	0.0115	0.0123	0.0110				
25	0.1636	0.1130	0.1226	0.0992	0.0677	0.0331	0.1244	0.1598	0.1022	0.1193			
28	0.1066	0.0659	0.0736	0.0690	0.0377	0.0249	0.0838	0.1136	0.0714	0.0863	0.0088		
29	0.0980	0.0805	0.0923	0.1589	0.0331	0.1037	0.0636	0.0786	0.0468	0.0462	0.0422	0.0358	
30	**0.0072**	0.0326	0.0227	0.1441	0.0379	0.1874	0.0097	0.0085	0.0197	0.0220	0.1762	0.1172	0.0987

*For instance, populations 5 and 30 were collected from two sites that were 80 km away from each other, while populations 11 and 24 had only 10 km distance. The geographic distances of the first group were 8 times further than the second, but the genetic distance was only 1/25.

### Multiple regression and Spearman rank correlations between environmental variables and SNPs

Principle components analysis (PCA) was carried out using the ecological factors as variables (as listed in Table 7, except for Sz, Ma, and So). The main ecological factors were selected for multiple regression analysis, which was mentioned in our previous paper [20]. Multiple regression analysis was done next using the ecological factors to investigate the relationship between environmental variables and SNPs. The geography, temperature, water, and solar radiation factors, singly or in combination, explained a significant proportion of the diversity in the SNPs (Table 8). The best variable predictors of *He *and *I *were two temperature factors: Tdd and Trd. The combination of geographic, temperature, and water availability factors were significant (p < 0.05) for genetic diversity (Table 8). SNPs in this gene could be classified into several categories associated with water, temperature, and geographic factors, respectively. The best single-variable predictors of SNP allele frequencies were: (1) water (Dw, Rr, Rv, Hu-14, and Th); (2) temperature (Tdd, Ta, and Sh); (3) geographic (Ln, Lt, and Al) factors (Table 8). It was obvious that water and temperature factors were the best variable predictors, singly or in combination, with other ecological factors (Table 8).

**Table 7:** The ecogeographical background of populations studied.

No.	Population	N	Ln	Lt	Al	Tm	Ta	Tj	Td	Tdd	Rn	Rd	Hu 14	Hu an	Dw	Sh	Th	Trd	Ev	Sz	Ma	So	Rv	Rr	Rad
5	Qazrin*	12	35.67	32.99	350	18	26	10	16	12	530	50	43	58	58	50	-	60	155	3	5	5	39	26	189
8	Gamla*	12	35.74	32.88	200	19	26	9	17	12	470	50	43	58	58	50	-	60	155	3	5	5	39	26	-
9	Rosh-Pinna	11	35.52	32.95	700	18	25	9	16	10	697	50	48	58	50	75	-10	35	150	3	5	1	35	22	184
11	Tabigha	22	35.53	32.90	0	24	32	15	17	10	436	45	45	57	58	60	-30	120	160	3	5	5	39	25	188
16	Mt. Gilboa	13	35.42	32.50	150	21	28	12	16	12	400	43	43	58	40	60	-30	160	165	2	3	1	34	24	189
17	Mt. Gerizim	14	35.28	32.20	800	17	23	8	15	9	700	45	45	60	42	-	10	0	155	2	3	1	38	25	186
18	Gitit	13	35.40	32.10	300	21	29	13	16	12	360	39	39	55	25	-	-25	100	170	2	3	1	38	24	195
19	Kokhav Hashahar	9	35.34	31.95	600	20	28	12	16	12	400	45	45	59	30	80	-20	25	165	2	3	1	38	22	195
23	Jaba	12	35.08	31.67	660	17	25	9	15	9	500	49	49	62	57	90	-20	30	155	2	3	1	35	21	186
24	Amirim	12	35.45	32.93	600	15	24	8	16	8	850	48	48	60	53	85	0	13	153	2	2	1	35	23	182
25	Nahef	9	35.32	32.93	275	15	24	8	15	9	670	49	49	62	57	62	10	3	155	1	2	1	33	22	181
28	Beit-Oren	16	35.03	32.73	400	17	24	11	13	8	700	59	59	69	80	41	5	0	142	1	2	1	25	19	183
29	Daliyya	8	35.06	32.59	200	19	26	12	14	11	670	57	57	67	78	50	-10	100	160	1	2	2	25	20	181
30	Bat-Shelomo	13	35.02	32.60	75	20	26	13	13	10	650	58	58	68	77	40	-10	30	150	2	2	2	24	20	182

* These two populations were collected from Golan near Qazrin and Gamla, respectively. Population numbers according to Nevo and Beiles 1989.

Symbols of Variables

i. **Geographical**: Ln = Longitude; Lt = latitude; Al = altitude

ii. **Temperature**: Tm = mean annual temperature; Ta = mean August temperature; Tj = mean January temperature; Td = seasonal temperature difference; Tdd = day-night temperature difference; Trd = mean number of tropical days; Sh = mean number of Sharav days, i.e., hot and dry days

iii. **Water availability**: Rn = mean annual rainfall; Rd = mean number of rainy days; Hu-an: = mean annual humidity; Hu-14 = mean humidity at 14:00 h; Dw = mean number of dew nights in summer; Th = Thornthwaite's moisture index; Ev = mean annual evaporation; Rv = mean inter-annual variability of rainfall; Rr = mean relative variability of rainfall

iv. **Edaphic**: So = soil type: 1 = terra-rossa (t.r.); 2 = rendzina; 5 = basalt

v. **Biotic**: Ma = marginality: 1 = North margin, 2 = West margin, 3 = South-east margin, 5 = central population; Sz = estimate of population size: 1 = small (from a dozen to few hundred plants), 2 = intermediate, 3 = large

**Solar radiation**: Rad = total solar radiation per year

**Table 8:** Coefficient of multiple regressions of genetic diversity, allele frequencies, and environmental variables in 14 populations of wild emmer wheat as independent variables.

Genetic indices	Stepwise model by ecogeographical variables					
	
	STEP1	STEP2	STEP3	STEP4	STEP5	FINAL STEP
*He*	Tdd 0.168ns	Trd 0.256ns				Trd 0.256ns
*I*	Tdd 0.141ns	Trd 0.219ns				Trd 0.219ns
**Allele Frequency**						
51G	Rv 0.296*	Tm 0.422*	Trd 0.611*			Trd 0.611*
72T	Tdd 0.221@	Tm 0.362@	Tj 0.426ns			Tj 0.426ns
84C	Tdd 0.183ns	Al 0.263ns				Al 0.263ns
96T	Rv 0.228@	Ev 0.400@	Rd 0.624*	Rn 0.796**	Trd 0.834**	Sh 0.896**
120G	Tdd 0.178ns	Trd 0.247ns				Trd 0.247ns
139G	Ta 0.195ns	Al 0.378@	Trd 0.465@			Trd 0.465@
141A	Tdd 0.157ns	Tm 0.352@	Tj 0.473@			Tj 0.473@
180G	Sh 0.239ns	Th 0.362@	Dw 0.433ns	Ta 0.554@	Tm 0.717*	Hu-14 0.786*
183C	Ta 0.232@	Al 0.344@	Rd 0.534*	Tdd 0.620*	Sh 0.704*	Rad 0.815@
228G	Hu-14 0.158ns	Ev 0.273ns	Rr 0.382ns	Tdd 0.464ns		Tdd 0.464ns
232G	Ta 0.232@	Al 0.344@	Rd 0.534*	Tdd 0.620*	Sh 0.704*	Rad 0.815@
246A	Tdd 0.194ns	Tm 0.408@	Rr 0.478@			Rr 0.478@
282T	Tdd 0.194ns	Tm 0.408@	Rr 0.478@			Rr 0.478@
288T	Tdd 0.194ns	Tm 0.408@	Rr 0.478@			Rr 0.478@
297G	Tdd 0.171ns	Tm 0.388@	Rv 0.460@			Rr 0.478@
315G	Tdd 0.135ns	Tm 0.317ns	Tj 0.468@			Rv 0.460@
336C	Tdd 0.178ns	Trd 0.247ns				Trd 0.247ns
338G	Ta 0.271@	Trd 0.462*	Al 0.540*			Al 0.540*
339G	Tdd 0.194ns	Tm 0.408@	Rr 0.478@			Rr 0.478@
350T	Al 0.172ns	Sh 0.334ns	Ta 0.627*	Rr 0.727*	Dw 0.855**	Th 1.000@
382T	Rv 0.318*	Hu-14 0.482*	Sh 0.536*			Sh 0.536*
396C	Lt 0.318*	Td 0.414@	Ln 0.521@	Rr 0.607@	Sh 0.653@	Ev 1.000*
415A	Tdd 0.178ns	Trd 0.248ns				Trd 0.248ns
433T	Sh 0.139ns	Al 0.217ns	Td 0.353ns	Ln 0.794**	Tm 0.918***	Rad 0.991***
438C	Rr 0.137ns	Rad 0.297ns	Dw 0.622*	Rv 0.676*	Td 0.722*	Td 0.722*
445C	Dw 0.180ns	Rr 0.408@	Ln 0.607*	Td 0.683*	Hu-an 0.805*	Hu-an 0.805*
451G	Ln 0.341*	Sh 0.490*	Tm 0.587*	Ev 0.869***	Rr 0.944***	Tj 0.973**
452T	Th 0.361*	Al 0.418@	Tm 0.511@	Ta 0.735*	Ln 0.888**	Lt 1.000**

*** = p < 0.001; ** = p < 0.01; * = p < 0.05; @ = p < 0.10;ns = p > 0.10.

Spearman rank correlations of ecological factors and genetic diversity of each of the SNP sites were shown in Table 8. Only one SNP was correlated with geographical factors (WMAI-451G was negatively correlated with Ln, rs = -0.521). Seven SNPs were positively or negatively correlated with the temperature factors of Tm, Ta, Td, Sh, and Tdd; another three SNPs were correlated with water (Table 9).

**Table 9:** Spearman rank correlations of ecological factors and genetic diversity of each SNP site (p < 0.10 level).

SNP	Ln	Tm	Ta	Tj	Td	Sh	Tdd	Rn	Rv	Rr	Hu-an	Hu-14	Rad
WMAI-51G	0.472	0.529	0.533*		0.541*		0.512	-0.476	0.619*		-0.592*	-0.482	
WMAI-84C							-0.459						
**WMAI-139G**													0.515
WMAI-180G						0.560*							
WMAI-183C			0.513	0.483			0.459						
**WMAI-232G**			0.513	0.483			0.459						
**WMAI-338G**		0.531	0.569*	0.513									0.519
**WMAI-382T**						0.566*			0.617*				
**WMAI-451G**	-0.521								-0.526	-0.579*	0.468		

** = p < 0.01; * = p < 0.05

## Discussions

### Genetic polymorphism of α-amylase inhibition sequences

The present study analyzed the extent of genetic polymorphisms and the effect of diversifying selection on SNPs in wild emmer wheat monomeric α-amylase inhibitor sequences among specific Israeli and Golan Heights' populations. This is the first time large numbers of emmer wheat accessions were sequenced for functional protein genes and natural selection is depicted on a modeled structure. Although SNP markers had already been used to assay the polymorphism of dimeric inhibitors, only 20% of SNP sites were successfully analyzed by the markers [21]. We obtained 384 WMAI gene sequences from 14 populations that belonged to 33 haplotypes, and 28 SNPs were detected (Table 1).

Different amino acid residues determined by polymorphic sites would significantly affect the structure, charge, and function of the inhibitors. The charge difference could be responsible for the relative mobility of gel electrophoresis and the differential inhibitory activities of α-amylase inhibitors [23]. In former investigations, more than five inhibitor proteins belonging to the WMAI family had been found, such as inhibitor 0.28, 0.32, 0.35, 0.39, and 0.48. They had similar molecular weights but different inhibiting activity [13]. The relationship between nucleotide polymorphisms and the amino acid changes in WMAI were summarized in Table 2. Only 11 SNPs in the nucleotide sequence of WMAI resulted in amino acid variations. Most polymorphic sites did not occur at the functional conservative site, which ensures the α-amylase inhibitors maintain their ability to combine with α-amylase. All cereal-type α-amylase inhibitors had 10 Cys (5 disulfide bonds). Both WMAI 0.28 and 0.39, similar to WDAI 0.19, were readily inactivated by treatments that break disulphide bonds, thus indicating that their stability mainly depends on the integrity of their disulphide bridges [24]. By combining FAB-MS and automatic sequencing, it was possible to assign the five disulfide bonds of the α-amylase inhibitor 0.28 from wheat kernels as follows: Cys7-Cys54, Cys21-Cys42, Cys29-Cys82, Cys43-Cys98, and Cys56-Cysl13 [25]. The monomeric α-amylase inhibitors from Israeli and Golan Heights' populations had 10 Cys (except for 7 accessions), and the positions of the 10 Cys were conserved. Most of the SNPs did not occur at highly conserved positions, which ensured that the α-amylase inhibitors would keep their correct 3D structure to combine with α-amylase.

The mutants obtained by García-Maroto et al. (1991) allow the identification of two regions of the molecule that are critical for inhibition mechanisms: the N-terminal sequence (positions 31-36; the signal peptide residues were 1-30) before the first Cys (Cys37) and the sequence after the seventh Cys, which is right after a CRC (positions 84-86) motif [26]. According to the alignment of deduced amino acid sequences of emmer wheat WMAI, no amino acids were changed by nucleotide mutations in this domain (Table 2). The majority of changes in amino acids occurred at the C-terminal, and most of the amino acids in the middle domain were conserved, ensuring the stability of WMAI (Table 2).

Although the most important positions were conserved, position analysis of the ratio of synonymous and non-synonymous substitutions provided strong evidence for natural selection acting on WMAI. The dN significantly differed from dS according to PAL2NAL results. However, the whole sequences dN/dS < 1 suggested that the inhibitors were under strong purifying selection pressure (indicating that there might be a structural requirement) and that amino acids at the C-terminal were positively selected; in other words, amino acid-altering substitutions offer fitness advantages that would result in the diversity of WMAI.

### Ecological Genetics and Evolution of WMAI

Experimental populations evolving under natural selection represent an interesting tool to study genetic bases of adaptation [27]. The ecological genetic analysis was carried out to investigate the evolutionary mechanism of WMAI from wild emmer wheat. The diversity of WMAI gene sequences from Israeli and Golan Heights' populations was revealed. Populations could be divided by SNPs, even within closely related populations originating in approximate geographic locations. Our results demonstrated that the polymorphism of monomeric α-amylase inhibitor genes in wild emmer wheat was correlated with the ecogeographic distribution of the accessions. Observations were consistent with previous results on other seed storage proteins such as HMW-GS and WDAI according to molecular markers [21,28-30].

Central populations used in this study were collected in warm, semi-humid environments on the Golan Plateau and near the Sea of Galilee. Marginal steppic populations were collected across a wide geographic area on the northern, eastern, and southern borders of wild emmer distribution involving hot, cold, and xeric peripheries; while marginally mesic (Mediterranean), populations were collected from the western border of wild emmer distribution [31]. The present study included 14 populations from different sites in Israel and Golan, and covered a wide range of ecogeographical conditions across the distribution range of the species. Specific SNP positions detected in WMAI were found highly effective in distinguishing genotypes and populations of wild emmer wheat originating from diverse ecogeographic sites in Israel and Golan. High levels of polymorphic loci (*P*), expected heterozygosity (*He*), and Shannon's information index (Table 5) with high genetic distance values between populations were found (Table 6). These results suggest that genetic variation at these SNP positions in the WMAI was at least partly ecologically determined for these populations.

The relationship between SNP genetic distance and geographical distance was investigated, and it was found that the estimates of genetic distance (*D*) were geographically independent. Sharp genetic divergence (large *D*) over very short geographic distances against small genetic divergence (small *D*) between large distances were observed, which was also found by allozymes, RAPD loci, SSR, and SNP marker analysis [21,32,33]. For example, the genetic distance between populations of Tabigha(11) and Amirim(24) (located only about 10 km apart with D = 0.1819) was 25 times higher than the genetic distance between populations Qazrin(5) and Bat-Shelomo(30) (separated by 80 km with D = 0.0072) (Figure 1).

Collection areas of wild emmer were different in altitude, longitude, latitude, and several other environmental factors (Table 7) [31]. It was noteworthy that SNPs in WMAI were correlated with ecological factors by multiple regressions and the Spearman rank correlations' matrix. Among the 28 SNPs, 11 SNPs led to amino acid changes at nine positions (Table 2). It was shown that these SNPs were significantly more correlated with water availability factors (Rv and Dw), temperature factors (Ta, Sh, and Tdd), and geographical factors (Ln, Al, and Lt) than the other factors (Table 8). Ecogeography, temperature, and water availability factors, singly or in combination, explained a significant proportion of the diversity in SNPs of α-amylase inhibitor genes. Diversity could be further explained by changes in ecological factors, i.e., Al (altitude), the sharp gradient of climatic conditions from north to south in Israel and Golan, with increasing temperatures and decreasing water availability towards the semiarid zones in southern Israel. Also, ecological factors taken into account for this study were not representative of all the possible components involved in the determination of the actual climate [32,33].

Environmental stress can greatly influence plant susceptibility to herbivores and pathogens, and drought stress can promote outbreaks of fungal diseases and plant-eating insects [34,35]. Herbivore insects and the level of herbivore pressure may vary with ecological factors. Different herbivore-related selection pressures at these ecological locations may influence polymorphism of insect-resistant loci in wild emmer wheat [21]. Different environmental pressures at each site related directly to the climate, but WMAI expression responded indirectly to environmental factors. It is possible that several evolutionary mechanisms underlie the differences in diversity of α-amylase inhibitors and ecological factors. It could be concluded that the variation in genetic diversity of the WMAI gene between populations is a product of selective forces.

The genetic structure of wild emmer wheat populations in Israel is mosaic [30]. This patchy genetic distribution appears to reflect the underlying ecological heterogeneity at micro- and macro-scales [32,33,36,37]. Thus, higher polymorphisms and genetic variations of WMAI within and between populations could be explained as adaptive complexes generated by natural selection and co-evolution with insects.

## Conclusions

Alpha-amylase inhibitors are attractive candidates for the control of seed weevils, as these insects are highly dependent on starch as an energy source. A total of 348 gene sequences of wheat monomeric α-amylase inhibitor (WMAI) were obtained; the frequency of SNPs was 1 out of 16.3 bases; 28 SNPs were detected in the coding sequence. Great diversity at WMAI loci, both between and within populations, was detected in the populations of Israeli and Golan Heights' wild emmer wheat. It was revealed that WMAI were naturally selected for across populations by the expected ratio of dN/dS. The results of purifying and positive selection hypothesis (p < 0.05) showed the sequences of WMAI were contributed by both natural selection and co-evolution, which ensures the conserved function as well as the inhibition of a variety of insect amylases. Ecological factors, singly or in combination, explained a significant proportion of the variations in SNPs. The conflict between genetic divergence and geographic distances also suggested that the SNPs in WMAI were subjected to natural selection, and ecological factors had an important evolutionary role in gene differentiation at this locus. These results suggested that α-amylase inhibitors are adaptively selected under different environments according to population and codon analysis.

## Methods

### Plant material and ecological background of wild emmer wheat

Wild emmer wheat (*T. dicoccoides*) is the wild progenitor of modern tetraploid and hexaploid wheat, which is predominantly a self-pollinated wheat distributed over the Near East Fertile Crescent [38]. A center of distribution and diversity of emmer wheat was found in the catchment area of the upper Jordan Valley in Israel and its vicinity [7]. Wild emmer wheat included 114 accessions from 14 populations, collected from various locations in Israel and Golan, which are representative of a wide range of ecological conditions such as soil, temperature, altitude, and water availability. Individual plants of emmer wheat were collected at random, at least 1 m apart, from populations differing in major ecological properties. These collection sites and populations have been described in detail in the literature [7,31]. The genotypes used for the present study are conserved in the cereal gene bank of the Institute of Evolution, University of Haifa. Populations used in this study along with their geographic origins and climatic conditions are listed in Table 7.

### DNA isolation and PCR amplification

Ten seeds of each accession were germinated in the dark at room temperature. Genomic DNA was extracted from plant leaves at about two weeks of age with a modified CTAB protocol as described in Murray and Thompson [39]. Specific primers (F: ATGTGGATGAAGACCGKGTT; R1: GACTAGRYGTCCGKATACGC; R2: CACGCACCGCACCATTACTT) for WMAI were used to amplify the gene coding sequences [40]. PCR amplification was performed with PTC-240 cycler (Bio-Rad) in a volume of 50 μL, which consisted of about 100 ng of genomic DNA, 100 μM of each dNTPs, 1 μM of each primer, 1U *Taq *polymerase, 1.5 mM Mg^2+ ^in 1 × PCR buffer. The cycling parameters were 95°C for 5 min to pre-denature, followed by 35 cycles of 95°C for 1 min, 55°C for 30 sec, and 72°C for 1 min with a final extension at 72°C for 5 min.

### SNPs mining and haplotype identification

Amplification products were separated in 2% agarose gels. Since the WMAI are encoded by multigenes, the desired DNA fragments were ligated to the pBluescript SK (+) T-vector plasmid (Stratagene), and then five positive clones were screened and sequenced.

The alignment of sequences and the SNP assessment were carried out using the multiple-sequence alignment software Clustal W http://www.ebi.ac.uk/clustalw and DNAman 5.2.2 http://www.lynnon.com. The α-amylase inhibitor ORFs were translated into amino acid sequences using the ORF Finder program at the NCBI http://www.ncbi.nlm.nih.gov. Polymorphic positions were identified by MEGA version 3.1 [41] and were used in place of all of the mutations' positions (the positions with change observed only once in the dataset were removed) in the subsequent analysis.

Subsequently, the alpha-amylase inhibitor genes from wild emmer wheat were analyzed by the median-joining network method [42], which was suitable to analyze the sequence from wheat and *Aegilops *species [43], and the phylogenetic clusters were demonstrated by using the program Network 4.5.1.0 http://www.fluxus-engineering.com/sharenet.htm. Since no phylogenetic study had been performed on alpha-amylase inhibitor gene types, a median-joining (MJ) network based on the sequence alignment of haplotypes was constructed.

### Selective pressure analysis

Codon-based Z-test to selection (p < 0.05) was carried out by using MEGA version 3.1 [41] to estimate nucleotide sequence divergence distances from synonymous and non-synonymous sites with the Nei-Gojobori model in standard error determined from 1000 bootstrap replicates. Ratios of non-synonymous substitutions per non-synonymous site to synonymous substitutions per synonymous site were computed for haplotypes of WMAI by PAL2NAL http://www.bork.embl.de/pal2nal/ using codon-based maximum likelihood methods contained in the codeml program of the software package PAML (dN/dS: dN/dS-value > 1 indicates positive selection, dN/dS < 1 purifying selection, and neutral evolution when dN/dS = 1) [44]. We also analyzed the selection of α-amylase inhibitor sequences with the program HYPHY http://www.hyphy.org version 1.002 beta [22]. FEL analyses were applied; all analyses employed the MG94 model of codon substitution. Results were considered significant when P value < 0.05. The sequence alignments and NJ tree were used to calculate the dN/dS (ω) ratio for each site.

### Ecological genetics analysis

The programs POPGENE 1.32, and STATISTICA 6.0 were used to carry out the ecological genetics analysis. POPGENE 1.32 [45] was used to analyze genetic polymorphism (*P*), expected heterozygosity (Nei's gene diversity) (*He*), and Shannon's information index (*I*) for each SNP position and population. STATISTICA version 6.0 http://www.statsoft.com/textbook/stathome.html was used to perform PCA analysis, stepwise multiple regression (MR), and Spearman rank correlation coefficients. Multiple regression analysis was conducted to test the best predictors using SNP frequencies as dependent variables and the ecogeographic factors as independent variables at each of the polymorphic SNP loci. Spearman rank correlation coefficients were used to assess differences in genetic indices *P*, *He*, and Shannon's information index in climatic variables for 14 populations.

## Authors' contributions

WJR designed the experiments, carried out the experiment, and wrote the manuscript. WYM planned the study and designed the experiment, formulated the question and contributed to writing the manuscript. DM participated in the experiment. NE formulated the question, retrieved and analyzed the data as well as planned the experiments. YZH carried out sequence alignment analyses. ZYL participated in the design of the experiments. All authors read and approved the final manuscript.
